# MediQuit – an electronic deprescribing tool: a pilot study in German primary care; GPs’ and patients’ perspectives

**DOI:** 10.1186/s12875-022-01852-2

**Published:** 2022-09-26

**Authors:** Matthias Michiels-Corsten, Navina Gerlach, Ulrike Junius-Walker, Tanja Schleef, Norbert Donner-Banzhoff, Annika Viniol

**Affiliations:** 1 Department of General Practice and Family Medicine, Faculty of Medicine, Philipps-University of Marburg, Marburg, Germany; 2 Institute for General Practice and Interprofessional Health Care, University Hospital Tübingen, Tübingen, Germany; 3 Institute of General Practice, Hannover Medical School, Hannover, Germany

**Keywords:** Polypharmacy, General practice/Family medicine, Primary Care, Multimorbidity, Health care technology, Shared decision-making / Patient involvement, Deprescribing

## Abstract

**Background:**

General practitioners (GPs) are the central coordinators for patients with multimorbidity and polypharmacy in most health care systems. They are entrusted with the challenging task of deprescribing when inappropriate polypharmacy is present.

MediQuit (MQu) is a newly developed electronic tool that guides through a deprescribing consultation. It facilitates the identification of a medicine to be discontinued (stage 1), a shared decision-making process weighing the pros and cons (stage 2), and equips patients with take-home instructions on how to discontinue the drug and monitor its impact (stage 3). We here aim to evaluate utility and acceptance of MQu from GPs’ and patients’ perspectives.

**Methods:**

Uncontrolled feasibility study, in which 16 GPs from two regions in Germany were invited to use MQu in consultations with their multimorbid patients. We collected quantitative data on demography, utility and acceptance of MQu and performed descriptive statistical analyses.

**Results:**

Ten GPs performed 41 consultations using MQu. Identification (step 1) and implementation elements (Step 3) were perceived most helpful by GPs. Whereas, shared-decision making elements (step 2) revealed room for improvement. Patients appreciated the use of MQu. They were broadly satisfied with the deprescribing consultation (85%) and with their decision made regarding their medication (90%).

**Conclusions:**

Implementation of MQu in general practice generally seems possible. Patients welcome consultations targeting medication optimization. GPs were satisfied with the support of MQu and likewise gave important hints for future development.

**Supplementary Information:**

The online version contains supplementary material available at 10.1186/s12875-022-01852-2.

## Background

Polypharmacy is widely accepted as a crucial risk factor in multimorbid and elderly patients.

It provokes adverse drug events, drug interactions, frailty, hospital admissions and mortality [1]. Deprescribing is a measure that tries to recover drug safety by questioning prescribing decisions when multiple prescriptions accrue into a complex and potentially hazardous drug regimen [2]. In this process of critical drug assessment, questionable medication is discontinued and subsequent effects are monitored. In most health care systems, deprescribing is the task that falls to GPs, who provide low threshold access and care coordination for multimorbid patients with polypharmacy [3]. German GPs generally recognize deprescribing as part of their ‘usual care’, but it mostly takes place occasionally with a lack of time and focus.

A variety of deprescribing tools have been developed to assist physicians in the complex environment of polypharmacy [4]. On the one hand, explicit lists of avoidable drugs in vulnerable patients offer support, such as Beers criteria [5], FORTA-list [6] and STOPP/Start criteria [7]. Algorithms based on more implicit criteria, on the other hand, make use of physicians’ judgement to identify avoidable drugs and offer a more generic approach to the problem of polypharmacy [4].

Despite the growing availability of such tools, deprescribing is still perceived with a multitude of challenges on the sociocultural, relational, organizational and individual level [3, 8, 9]. Electronic tools promise to overcome some of these challenges. They are able to integrate complex information utilizing patient health data and prescribed medications on the one hand and guideline recommendations as well as pharmaceutical caveats on the other hand [10–12].

However, supporting only physicians would be too narrow. Shared decision-making (SDM) and patient-centred approaches should be deployed in all areas of health-care [13]. This also applies to deprescribing decisions. Furthermore, this approach allows sharing of uncertainty too and may so help in overcoming barriers. Conveying complex medical deprescribing considerations to elderly patients in an easy-to-understand manner is as demanding as elucidating patients’ preferences and treatment goals to ultimately incorporate both perspectives into a shared decision [14]. So far, none of the existing tools sufficiently manages to integrate a patient-centred approach into a medication review and—if deemed necessary – into a deprescribing approach [4].

For this reason, we developed arriba MediQuit (MQu). It is an electronic tool, that encourages GPs and their patients to rendezvous in a special ‘deprescribing consultation’. Here, patients and physicians review and assess the patients’ medication regimen and identify, weigh, and decide on opportunities for improvement. First evaluation of MQu by our team indicate satisfactory effectiveness with 70% of patients reporting drug discontinuation [15].

In contrast to the previous evaluation [15], the analysis presented here aims to examine the perceived usefulness, uptake and acceptance of MQu as a deprescribing tool from GPs’ as well as patients’ perspectives. In addition, we here offer a more detailed insight into technological structure und visualization of MQu.

## Methods

### Design

Between December 2018 and July 2019, we conducted a single group feasibility pilot study in primary care practices in two regions of Germany (Lower Saxony and Hesse).

### Study population

Sixteen GPs were recruited using convenience sampling from our academic primary care research networks. Patients were recruited by their GPs consecutively when meeting eligibility criteria. GPs were asked to include three to five patients each. To be eligible for study inclusion, patients had to be at least 60 years of age, take five or more long-term medications and have a minimum of three chronic diseases. Patients with deficient language proficiency or with severe cognitive impairment were excluded. Since MQu was not yet compatible with mobile devices, patients seen on home or nursing home visits only, were excluded.

### Ethics

The ethics committees of Hannover Medical School (No 2326–2014) and Marburg University (No 160/15) gave approval to the study. GPs gave written informed consent after written and oral explanations of the study procedure and its objective. Patients were informed by their GP about the study objectives and that during their consultation a computer tool is used to identify potential optimizations of their drug regimen. Patients gave written informed consent.

### Study procedure

Each practice was initially instructed on the study procedure and documentation requirements via visits of a research team member. Practice nurses were shown how to pre-select suitable candidates, collect pre-specified patient data, and perform documentation using protocols. GPs additionally received individual training sessions on how to apply MQu that was installed on local hardware. Training included familiarisation with its three-step structure and its information and visualisation features, as well as practical application using a clinical case vignette. After initiation, practices started including patients on a continuous basis. GPs subsequently conducted MQu-assisted medication counselling in their practices. They rendered patient baseline-data and documented their experiences using case reporting forms.

### Description of the MediQuit tool

The developmental process was based on results of a systematic literature review [4], our own clinical experience, and iterative discussions within the research team. Additionally, ten external experts from different fields (pharmacology, polypharmacy, primary care guidelines, geriatric medicine, multimorbidity, technology, public health, and shared decision-making (SDM)) were asked to comment on the tool. We decided to use the arriba platform, since this is an already broadly disseminated clinical decision support system (CDSS) used for shared decision-making in various contexts (e.g. risk-estimation in cardio-vascular prevention) in German primary care [16]. We designed an arriba module (MQu) supporting deprescribing and guiding through three consecutive steps.

1. The first step assists with the identification of potentially unnecessary or inappropriate drugs. GPs preselect one of the patient’s drug they medically assume most suitable for deprescribing. In the following, this target-drug is assessed with help of the tool. The tool’s assistance entails a criterion-based six-step algorithm, probing for the presence of drug indication, mode of action (symptomatic vs preventive), subjective benefit, effectiveness and safety, goals of care as well as medication related problems. The criteria were linked into an “if/then” algorithm basing on a tailored hierarchical sequence of drug evaluation. The criterion-based medication assessment is not automated. Treatment and patient information are assumed familiar to the physician or, if needed, elicited under patient counselling. Further, MQu provides explicit lists of avoidable drugs, if required.
  Succeeding completion of the first step, MQu generates one of three possible recommendations, namely to a) deprescribe b) reduce dosing or change medication or c) continue the assessed drug. This is illustrated using a traffic light labelling.
2. The second step of MQu promotes exchange of information between physician and patient in a shared decision-making (SDM) process. MQu offers both, visual and communication-based tools for an informed physician–patient interplay. Communication cues are offered to GPs to assist the consultation. Further, individualized arguments for and against continuation of drugs are positioned in a weighing scale.
3. The shared decision on how to proceed with the discussed drug leads to the third step. Here, MQu assists with implementing a medication optimization plan. Several supporting elements are offered to assist with the medication change, dose reduction or deprescribing. Finally, a printout template is offered in which physicians may render detailed tapering instruction, possible adverse drug withdrawal events (ADWEs), ways to counteract those, and schedule follow-up visits.
  The idea was that patients leave the consultation with a printout summarizing the discussed changes and empower patients for self-monitoring. GPs were also requested to handout an updated medication schedule in parallel. A follow-up appointment was offered, if deemed necessary.

Screenshots and a video of MQu are available on https://arriba-hausarzt.de/module/mediquit (English screenshots supplementary file 1).

### Data collection and analysis

After each MediQuit consultation, GPs were asked to complete two case reporting forms (CRF):

- CRF 1 asked for patients’ health data: frailty status according to the study of osteoporotic Fractures (SOF) [17], list of patients’ diagnoses and medication scheme before the intervention.
- CRF 2 asked for ratings of the consultation and the tool: name of medication checked, (shared) decision-making including its result, duration of the consultation, MQu elements used, helpfulness of elements, evaluation of benefit, free comments for improvements.

Additionally, every GP was asked to fill a final questionnaire after completion of all consultations rating the tool’s comprehensibility, helpfulness, satisfaction with elements, patient-involvement (SDM), and intention for future use.

Patients were followed up by telephone at T1 (2–4 days after consultation) und T2 (4 weeks later). They were asked to name the medication(s) discussed, prior trials of withdrawal, result of consultation, their involvement (understandability, room for questions, decision-making, satisfaction), implementation (discontinuation, occurrence of health issues), assessment of duration, evaluation of print-out, general assessment of medication consultation and tool.

Data were imported into IBM SPSS Statistics 26 and analysed descriptively.

## Results

### Sociodemographic data

Sixteen GPs were enrolled in the study (eight each in Hesse and Lower Saxony). Six of them were not able to perform medication counselling with the help of MQu, because of organizational or time constraints, language barriers, or technical issues. Ten GPs recruited patients and performed medication counselling with the help of MQu (see Table 1 for characteristics of actively recruiting GPs).

**Table 1:** GP characteristics (*n* = 10)

**GPs (*n* = 10)**	Total *n* = 10
Age, mean: years (range)	48 (33–60)
Gender n	
Female	6
Experience in practice: mean years (range)	20 (7–33)
Practice n (%)	
Single-handed	4 (40)
Group-practice	6 (60)
Setting^a^ n (%)	
Rural	3 (30)
Small town	3 (30)
Medium-sized town	0
Urban	4 (40)

^a^ Setting of practice: rural < 5,000, small town 5,000–20,000, medium-sized town 20,000–100.000, urban > 100,000 inhabitants

GPs conducted consultations using MQu with a total of 41 patients, thereof 16 men and 25 women, mean age 77 years with a mean number of 10.9 prescribed medications (see Table 2).

**Table 2:** Patient characteristics (*n* = 41)

**Patients**	Total *n* = 41
Age in years: mean (S.D.)	77.1 (8.1)
Sex n (%)	
Female	25 (61)
Known to practice in years: mean (S.D.)	11.6 (11.5)
Number of diagnoses mean (S.D.)	8.2 (2.2)
Frail patients according SOF^a^ n (%)	12 (30)
Patients on care dependency^b^ n (%)	8 (20)
Number of prescribed medication^c^ mean (S.D.)	10.9 (3.8)

Legend

^a^ Frailty test according to Study of Osteoporotic Fractures (SOF) [17]

^b^ Care dependency status classified by the health insurance fund

^c^ including on demand medication

Of the 41 patients, 37 participated in T1-follow-up (2–4 days), and 35 patients participated in T2-follow-up (4 weeks). Patients lost to follow-up could not be contacted by telephone (3), withdrew consent (2) or were medically not able to participate in follow-up interview (1).

All GPs were familiar with the arriba platform in advance, and 7 of 10 GPs used the platform regularly (all used arriba module for cardiovascular prevention). The arriba MQu module was introduced to GPs for the first time during this study.

### Duration of consultation

In mean, deprescribing consultations lasted 15 min (Interquartile range IQR 10–20 min). The majority (87%) of consultations were perceived of perfect duration by GPs. Fifteen percent were perceived as too long. Patients in turn, marked 95% of consultations as perfect in length, whereas 5% of patients considered consultations as too short.

### Usage and helpfulness of MQu

GPs indicated to have used the MQu steps 1 and 3 frequently (71–87%) (s. Table 3).

**Table 3:** Usage of MQu elements during 41 patient consultations

MQu Step	Element	Usage of element n (%)
1 Identification of target drug	Identification- criteria (*n* = 39^a^)	36 (88)
	Traffic-lights (*n* = 40)	36 (88)
2 Shared decision-making	Scales (*n* = 40)	12 (29)
	Communication cues (*n* = 39)	13 (32)
3 Implementation	Deprescribing schedule (*n* = 40)	31 (76)
	Patient print-out (*n* = 40)	29 (71)
1–3 Additional information	Information boxes (*n* = 40)	19 (46)
	Linkages to external sources (*n* = 39)	3 (7)

^a^ This No indicates, how many GPs have replied on this question, e.g. 39 replies, hereof 36 have used the element, 3 did not use the element, 2 replies were missing

Whereas, shared decision-making elements in step 2 as well as information boxes and external sources were used less often (7–46%).

**Fig. 1:**
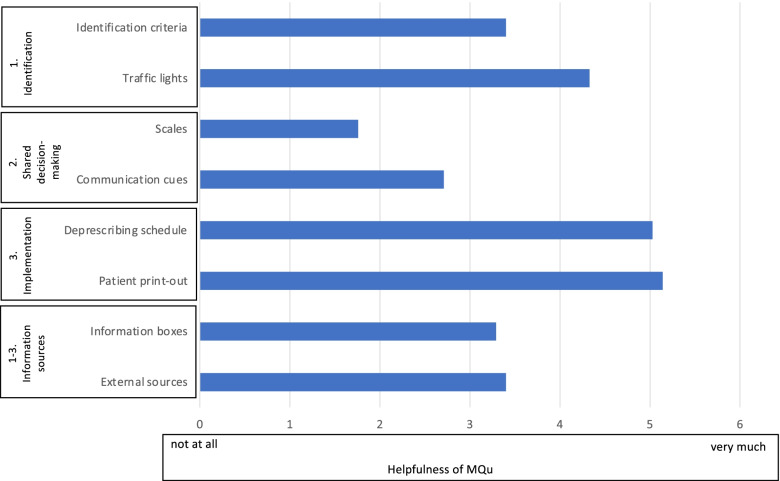
Helpfulness of MQu elements for GPs during deprescribing counselling (*n* = 41); Mean rating on Likert Scale 0 (not at all) to 6 (very much)

When GPs were asked on how helpful MQu elements were during counselling, the elements of step 3 implementation (patient print-out and deprescribing schedule) were perceived as most helpful (Fig. 1). Whereas, elements of step 2 developed to promote shared decision-making (communication cues and scales) were perceived as least helpful.

### General evaluation

When GPs were finally asked for their evaluation after all of their consultations with MQu, they found the program generally suitable for their patients (70%) and would be most suitable for elderly patients on multiple drugs with complex regimes.

**Fig. 2:**
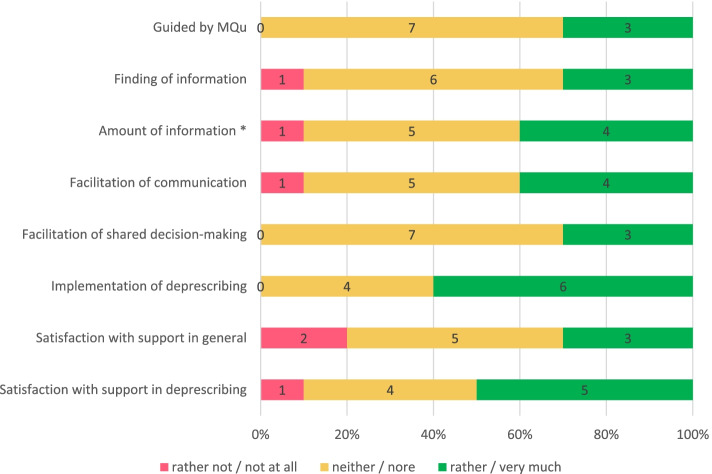
GPs Satisfaction with MQu (*n* = 10, numbers indicating relative proportion of GPs x/10). * In “Amount of information” the rating corresponds to: too little (1), too much (5) and perfect (4)

In general, GPs reported that MQu supported communication with patients rather or very much in 40% (Fig. 2). While 70% of GPs reported the decision had been a shared one, only 49% of patients did report so (data not shown). However, SDM elements were seen supportive by 30% of GPs, whereas the implementation of the deprescribing regime was experienced supportive by 60% (Fig. 2).

In general, 50% of GPs were satisfied with the assistance of MQu in deprescribing.

GPs made various suggestions for improvements mainly referring to a more intuitive and clearer design, adjustable SDM components (scale and communication cues) for more individual counselling, and embedding of specific drug as well as guideline recommendations.

When asked for their intention for future use, GPs appeared highly encouraged to pick up MQu step 3 (implementation) in future consultations. The use of SDM elements (step 2) revealed least attractive. This goes in parallel with GPs’ satisfactions with MQu as well as their suggests for improvements mentioned above.

### Patients’ perspective

Patients generally appreciated the medication consultation targeting deprescribing (80%). The use of an electronic tool (MQu) was generally considered positive in the majority of cases (63%). Patients reported general satisfaction with the deprescribing consultation (85%) as well as satisfaction with their decision made (90%).

When asked for MQu, 51% indicated to have looked at the MQu screen during consultation (Table 4), whereby most were not able to specify or did not remember what they had seen. Given information during counselling was perceived rather and completely comprehensive in most cases (85%), and most patients described enough room for questions (83%). Twentyfive patients (61%) received a print out, what was read by about half of them (Table 4). One third of patients indicated to be willing to check their mediation for deprescribing again with MQu, preferably with a focus on antihypertensives and statins.

**Table 4:** Patients perspective on MQu counselling (*n* = 41)

		**Yes**	**No**	**Missing**
**Looked at MQu screen**		**21**	**16**	**4**
**Information comprehensive**		**35**	**1**	**5**
**Room for questions**		**34**	**0**	**7**
**Print-out**	**received**	**25**	**12**	**4**
	**read**	**17**	**8**	**16**
	**understood**	**17**	**0**	**24**
	**information enough**	**13**	**4**	**24**

## Discussion

### Main findings

Arriba MediQuit (MQu) is a novel electronic three step tool, developed to support GPs and their patients in consultations dealing with polypharmacy and deprescribing. It was tested with ten GPs in 41 consultations. Here, we offer a detailed insight into technological parameters and vizualization of MQu, for the first time. GPs rated identification (step 1) and implementation support (step 3) most helpful and showed intention for future use of these elements, when asked for an overall evaluation of the tool. Whereas, SDM elements and auxiliary information sources revealed room for improvement. Patients generally appreciated consultations and the use of MQu. While they were broadly satisfied with the deprescribing consultation and their decision made, only half of the patients looked at the MQu screen and about two thirds received a printout.

### Strengths and limitations

A strength of this pilot study lies in its pragmatic design in two German regions, with urban as well as rural practices and with a diverse group of GPs. Most of the GPs were able to successfully integrate a MQu deprescribing consultation into their surgery. However, even in this small sample, six of 16 GPs did not manage to test MQu with their patients. GPs reported obstacles in finding time to familiarise with MQu, technical difficulties in installing and handling the program as well as language barriers with some patients. Due to the usual limitations in the design of pilot studies (short follow up, potential selection bias, no blinding, no control and no randomisation), we cannot yet make a statement on the actual efficacy of MQu. Nevertheless, the elementary design helped us generating pivotal results for future development of MQu and other tools in the context. Furthermore, the simple approach to meet in an extra appointment (with or without MQu) may alone improve the uptake of deprescribing efforts.

To our knowledge, arriba MediQuit (MQu) is the first electronic deprescribing tool that integrates SDM. However, the helpfulness in communication and patient-involvement were shown to need further improvements. There still seem to be some obstacles to make advantage of SDM in this context. The minority of patients looked at the MQu screen or remembered what they had seen.

Notwithstanding, patients showed high willingness to participate in the study and discuss medication optimization with their GP. They were highly satisfied with the consultation, the use of MQu and their decisions made. Some even wished further consultations with MQu. The majority reached a sustained discontinuation of discussed drugs [15].

The missing ability to extract information from electronic health records (EHR) was another point of critics by GPs. Whereas, identification and implementation elements of MQu were perceived extremely helpful by them.

### Comparison with literature

In contrast to other electronic tools (as TRIM [10], Prima-eDS [11], TaperMD [18]), MQu has no automated drug assessment, does not automatically align EHR data (laboratory, comorbidities, medication interaction etc.) with clinical guidelines and thus does not automatically generate advice for changing drugs.

MQu, on the contrary, requires the skilled input and evaluation by GPs. MQu assists GPs where support is needed. The reliance on the GP’s judgement avoids time-consuming data input (up to 20 Min in Prima-eDS [19]). But even more importantly, this opens the space for GPs and their patients to discuss the topic and find individual solutions based on the patients’ personal goals of care.

Janssen et al. stress the importance of SDM in deprescribing [20]. Their ‘analysis’ offers advice and narrative suggestions in this context. In parallel with the SDM literature, we found conflicting ratings about the presence of SDM between patients and GPs [21]. However, as far as we know, MQu is the only electronic tool that focuses on this topic and presents special support in communication and shared-decision making.

The integration of explicit lists of avoidable medication is not integral part of MQu in contrast to other tools. Since their explicit character might be helpful in some circumstances, we integrated links to useful projects like Medstopper [22] and others.

Scott and Couteur (2015) are right when calling (hospital) physicians for active deprescribing [23]. On the other hand, patient empowerment falls short in this respect. By elucidating patients’ preferences and involving patients in decision, we want patients to take the lead in deprescribing.

In line with the literature, patients were open in this study to discuss their medication and discontinue drugs [24]. Patients even wish to be more involved [24].

Implementation of deprescribing approaches revealed multiple obstacles [25]. MQu was able to overcome some of them, while support in implementing of deprescribing intentions was rated most helpful.

### Implications for research and practice

On the one hand, this study once again demonstrates that deprescribing is possible and that GPs as well as patients welcome the approach to discuss and decide on polypharmacy. Deprescribing in primary care is feasible and further development worth its effort.

On the other hand, we had to realise that communication and SDM in MQu need improvements in terms of a more intuitive and more individualizable design. GPs need a real interactive screen that itself stimulates interaction and discussion of the parties. Just by a true involvement of patients, a real sharing of information and decisions becomes achievable.

In this trial, GPs were asked to go through all components of MQu sequently. This seemed too inflexible for their needs. GPs wished for a more flexible tool that selectively supports, where support really is needed.

Further development has to go along with technical improvements as well. An easily accessible platform that runs on portable devices is strongly needed. Integration of an interface to communicate with existing HER could further open up opportunities.

Finally, the improved tool needs a robust validation in an effectiveness RCT.

## Conclusions

Implementation of MQu in daily GP routine generally seems possible. Patients were open to medication optimization and deprescribing. They were satisfied with their decisions made. GPs indicated sufficient use of most elements and likewise gave important suggestions for future developments.

## Supplementary Information

**Additional file 1.**

## Data Availability

The datasets used and analyzed during the current study are not publicly available due to missing anonymizations and lack of approval by authors and participants for unbounded publication, but are individually available from the corresponding author on reasonable request.

## Abbreviations

GP: General practitioner
MQu: arriba MediQuit software
CDSS: Clinical decision support system
SDM: Shared decision-making
ADWE: Adverse drug withdrawal event
HER: Electronic health records

## References

[1] Wang R, Chen L, Fan L, Gao D, Liang Z, He J (2015). Incidence and effects of polypharmacy on clinical outcome among patients aged 80+: a five-year follow-up study. PLoS One.

[2] Reeve E, Shakib S, Hendrix I, Roberts MS, Wiese MD (2014). Review of deprescribing processes and development of an evidence-based, patient-centred deprescribing process. Br J Clin Pharmacol.

[3] Gerlach N, Michiels-Corsten M, Viniol A, Schleef T, Junius-Walker U, Krause O (2020). Professional roles of general practitioners, community pharmacists and specialist providers in collaborative medication deprescribing - a qualitative study. BMC Fam Pract.

[4] Michiels-Corsten M, Gerlach N, Schleef T, Junius-Walker U, Donner-Banzhoff N, Viniol A (2020). Generic instruments for drug discontinuation in primary care: A systematic review. Br J Clin Pharmacol.

[5] By the 2019 American Geriatrics Society Beers Criteria® Update Expert Panel. American Geriatrics Society 2019 Updated AGS Beers Criteria® for Potentially Inappropriate Medication Use in Older Adults: 2019 AGS BEERS CRITERIA® UPDATE EXPERT PANEL. J Am Geriatr Soc. 2019. 10.1111/jgs.15767.

[6] Pazan F, Weiss C, Wehling M, FORTA (2018). The EURO-FORTA (Fit fOR The Aged) List: International Consensus Validation of a Clinical Tool for Improved Drug Treatment in Older People. Drugs Aging.

[7] O’Mahony D, O’Sullivan D, Byrne S, O’Connor MN, Ryan C, Gallagher P (2015). STOPP/START criteria for potentially inappropriate prescribing in older people: version 2. Age Ageing.

[8] Wallis KA, Andrews A, Henderson M (2017). Swimming Against the Tide: Primary Care Physicians’ Views on Deprescribing in Everyday Practice. Ann Fam Med.

[9] Doherty AJ, Boland P, Reed J, Clegg AJ, Stephani A-M, Williams NH (2020). Barriers and facilitators to deprescribing in primary care: a systematic review. BJGP Open.

[10] Fried TR, Niehoff KM, Street RL, Charpentier PA, Rajeevan N, Miller PL (2017). Effect of the Tool to Reduce Inappropriate Medications (TRIM) on Medication Communication and Deprescribing. J Am Geriatr Soc.

[11] Rieckert A, Reeves D, Altiner A, Drewelow E, Esmail A, Flamm M, et al. Use of an electronic decision support tool to reduce polypharmacy in elderly people with chronic diseases: cluster randomised controlled trial. BMJ. 2020;369:m1822. https://www.bmj.com/content/369/bmj.m1822. 10.1136/bmj.m1822 PMC7301164 32554566

[12] McDonald EG, Wu PE, Rashidi B, Forster AJ, Huang A, Pilote L (2019). The MedSafer Study: A Controlled Trial of an Electronic Decision Support Tool for Deprescribing in Acute Care. J Am Geriatr Soc.

[13] Ferrer  L (2015). Engaging patients, carers and communities for the provision of coordinated/integrated health services: strategies and tools. World Health Organ Reg Off Eur.

[14] Thompson W, Le JV, Haastrup P, Nielsen JB, Pedersen LB, Jarbøl DE (2020). Exploring how GPs discuss statin desprescribing with older people: a qualitative study. BJGP Open.

[15] Junius-Walker U, Viniol A, Michiels-Corsten M, Gerlach N, Donner-Banzhoff N, Schleef T (2021). MediQuit, an Electronic Deprescribing Tool for Patients on Polypharmacy: Results of a Feasibility Study in German General Practice. Drugs Aging.

[16] arriba. arriba Hausarzt. https://arriba-hausarzt.de/. Accessed 28 Jan 2022.

[17] Ensrud KE, Ewing SK, Taylor BC, Fink HA, Stone KL, Cauley JA (2007). Frailty and Risk of Falls, Fracture, and Mortality in Older Women: The Study of Osteoporotic Fractures. J Gerontol Ser A.

[18] McMaster University (2020). eam Approach to Polypharmacy Evaluation and Reduction TAPER Randomized Controlled Trial. Clinical trial registration. clinicaltrials.gov.

[19] Rieckert A, Sommerauer C, Krumeich A, Sönnichsen A (2018). Reduction of inappropriate medication in older populations by electronic decision support (the PRIMA-eDS study): a qualitative study of practical implementation in primary care. BMC Fam Pract.

[20] Jansen J, Naganathan V, Carter SM, McLachlan AJ, Nickel B, Irwig L (2016). Too much medicine in older people?. Deprescribing through shared decision making BMJ.

[21] Drivenes K, Haaland VØ, Hauge YL, Vederhus JK, Irgens AC, Solli KK (2020). Discrepancy in Ratings of Shared Decision Making Between Patients and Health Professionals: A Cross Sectional Study in Mental Health Care. Front Psychol.

[22] James McCormack et al. MedStopper.com. http://medstopper.com/. Accessed 28 Jan 2022.

[23] Scott IA, Couteur DGL (2015). Physicians need to take the lead in deprescribing. Intern Med J.

[24] Lundby C, Glans P, Simonsen T, Søndergaard J, Ryg J, Lauridsen HH (2021). Attitudes towards deprescribing: The perspectives of geriatric patients and nursing home residents. J Am Geriatr Soc.

[25] Ailabouni NJ, Nishtala PS, Mangin D, Tordoff JM (2016). Challenges and Enablers of Deprescribing: A General Practitioner Perspective. PLoS One.

