# Supplementary material for: MediQuit – an electronic deprescribing tool: a pilot study in German primary care; GPs’ and patients’ perspectives
Source: BMC Prim Care. 2022 Sep 26;23:252. doi: 10.1186/s12875-022-01852-2 (PMC9511770; doi:10.1186/s12875-022-01852-2)
Supplement: Supplementary file 1 — Additional file 1. [file 12875_2022_1852_MOESM1_ESM.pdf]

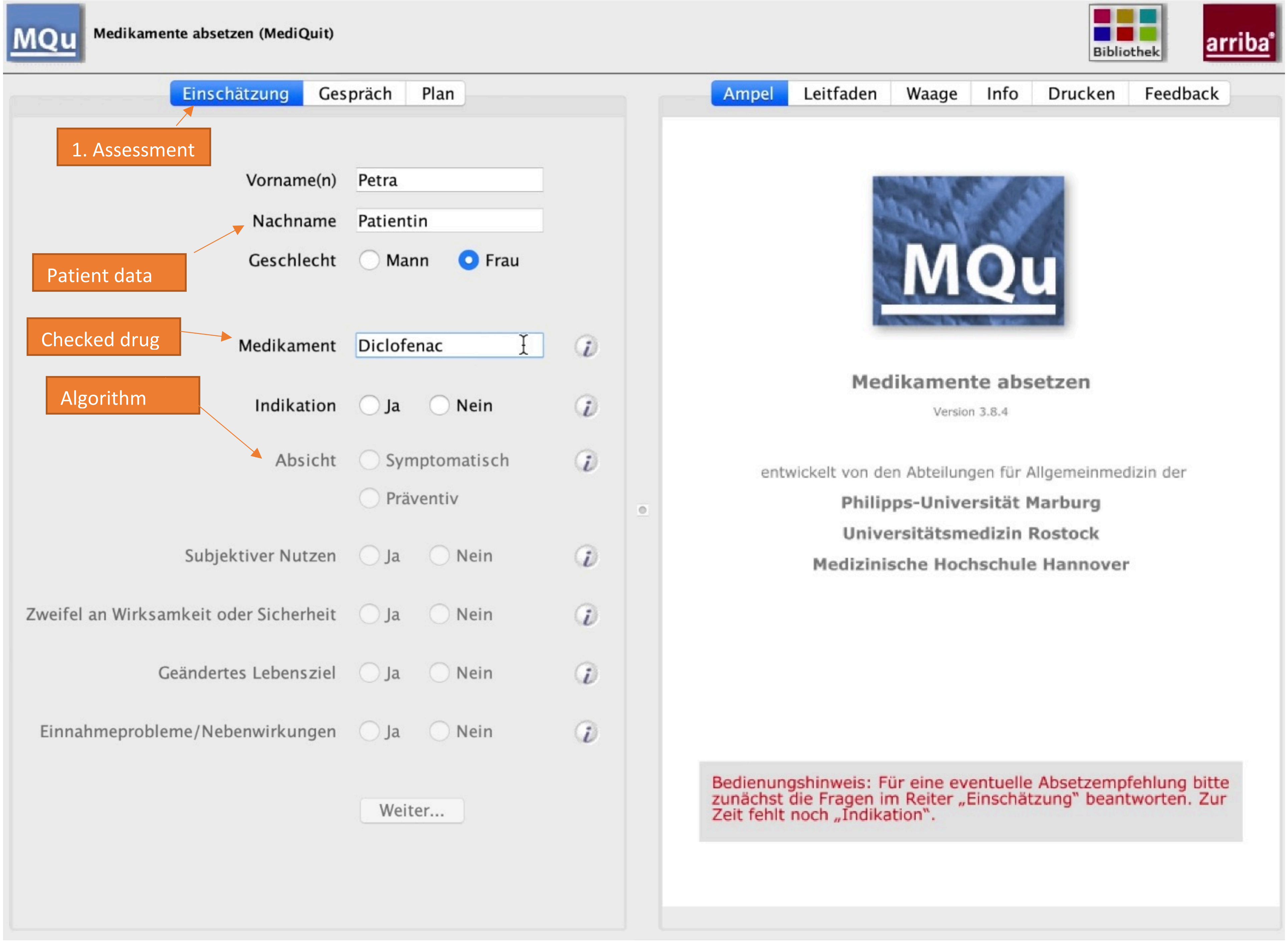
MQu
Medikamente absetzen (MediQuit)
Bibliothek
arriba®
Einschätzung
Gespräch
Plan
1. Assessment
Vorname(n) Petra
Nachname Patientin
Patient data
Geschlecht Mann Frau
Checked drug
Medikament Diclofenac
Algorithm
Indikation Ja Nein
Absicht Symptomatisch Präventiv
Subjektiver Nutzen Ja Nein
Zweifel an Wirksamkeit oder Sicherheit Ja Nein
Geändertes Lebensziel Ja Nein
Einnahmeprobleme/Nebenwirkungen Ja Nein
Weiter...
Ampel
Leitfaden
Waage
Info
Drucken
Feedback
MQu
Medikamente absetzen
Version 3.8.4
entwickelt von den Abteilungen für Allgemeinmedizin der
Philipps-Universität Marburg
Universitätsmedizin Rostock
Medizinische Hochschule Hannover
Bedienungshinweis: Für eine eventuelle Absetzempfehlung bitte zunächst die Fragen im Reiter „Einschätzung" beantworten. Zur Zeit fehlt noch „Indikation".

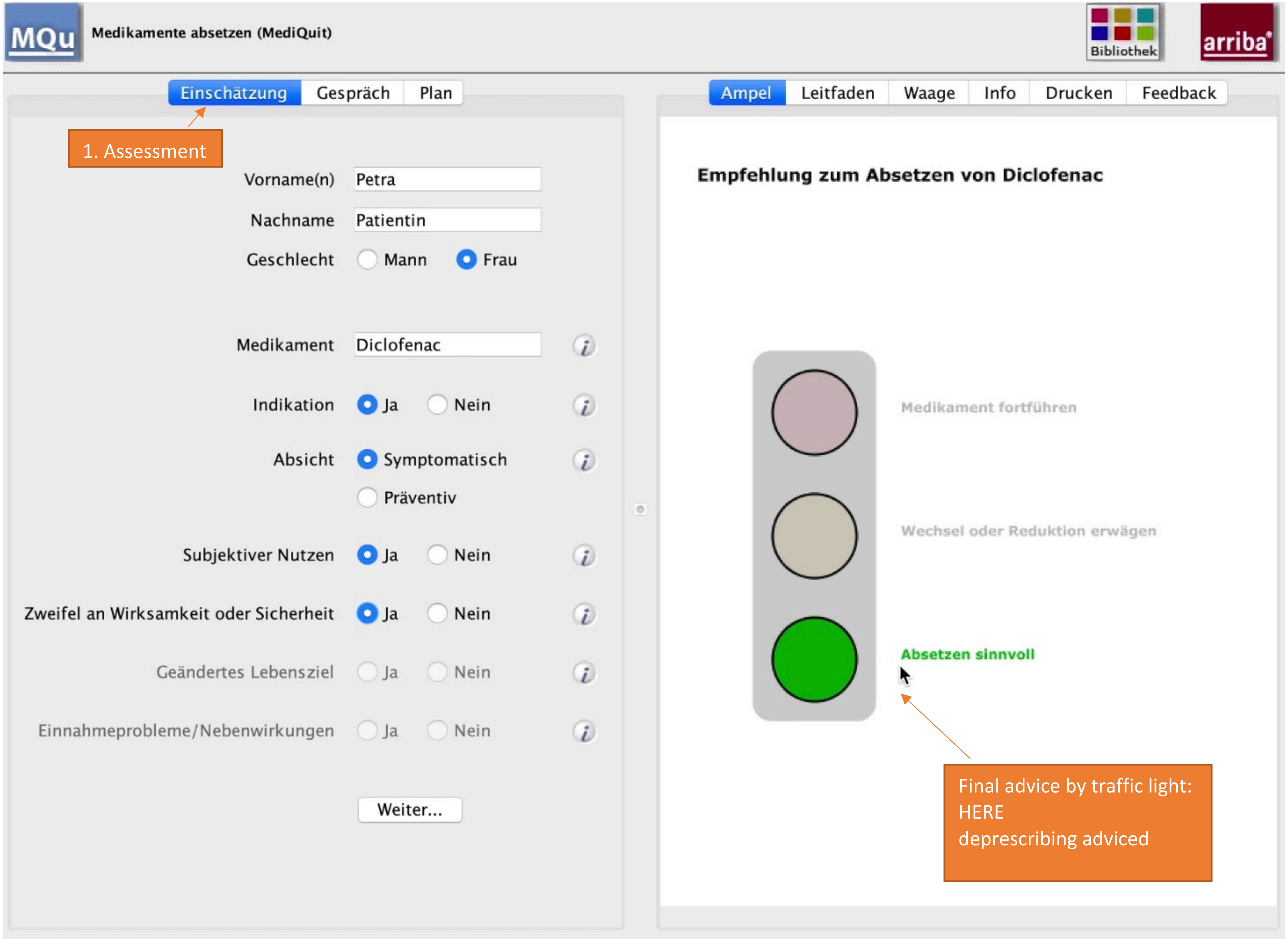
MQu
Medikamente absetzen (MediQuit)
Bibliothek
arriba
Einschätzung
Gespräch
Plan
1. Assessment
Vorname(n)
Petra
Nachname
Patientin
Geschlecht
Mann
Frau
Medikament
Diclofenac
Indikation
Ja
Nein
Absicht
Symptomatisch
Präventiv
Subjektiver Nutzen
Ja
Nein
Zweifel an Wirksamkeit oder Sicherheit
Ja
Nein
Geändertes Lebensziel
Ja
Nein
Einnahmeprobleme/Nebenwirkungen
Ja
Nein
Weiter...
Ampel
Leitfaden
Waage
Info
Drucken
Feedback
Empfehlung zum Absetzen von Diclofenac
Medikament fortführen
Wechsel oder Reduktion erwägen
Absetzen sinnvoll
Final advice by traffic light:
HERE
deprescribing adviced

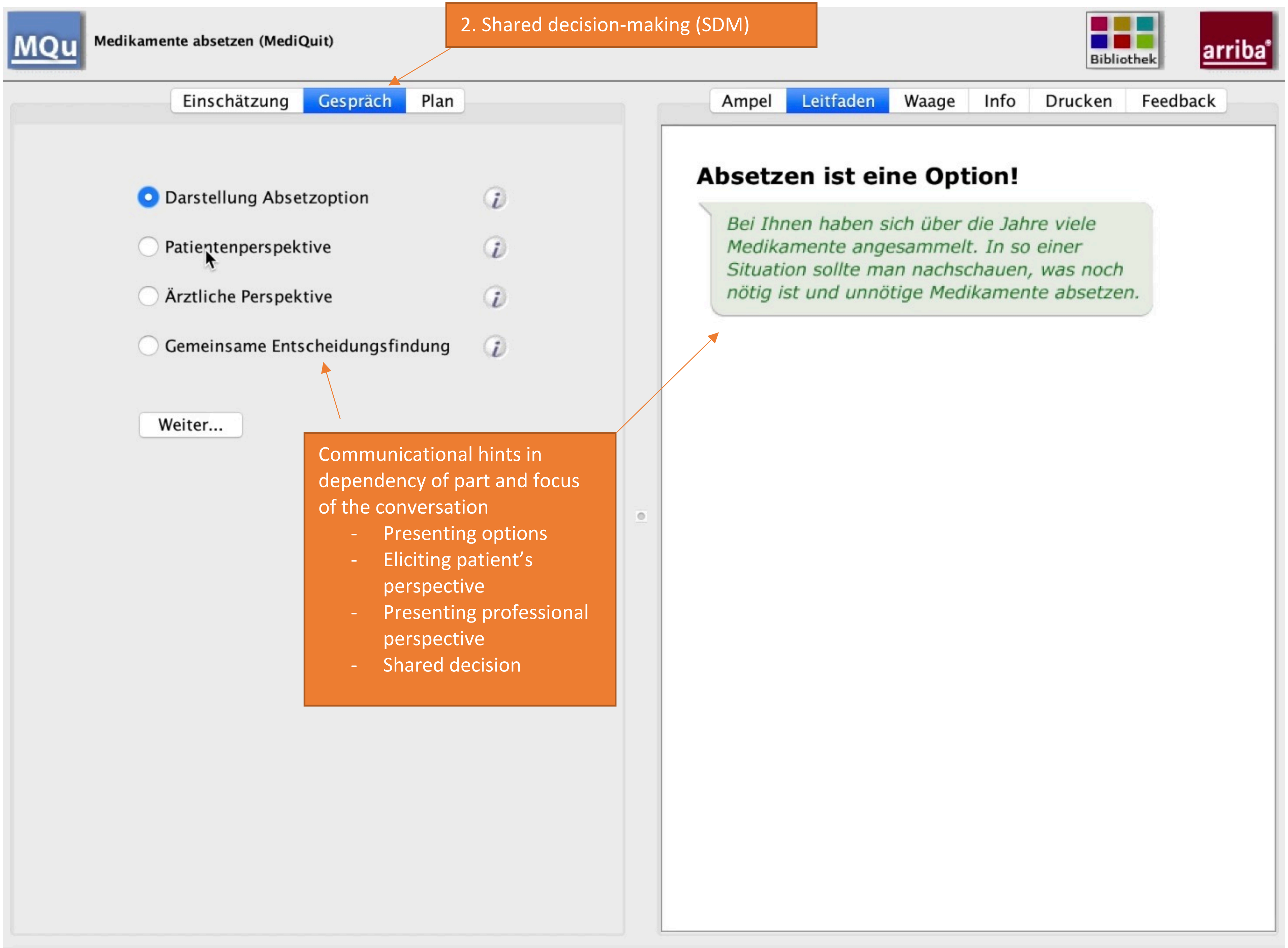

MQu
Medikamente absetzen (MediQuit)
2. Shared decision-making (SDM)
Bibliothek
arriba
Einschätzung
Gespräch
Plan
Ampel
Leitfaden
Waage
Info
Drucken
Feedback
Darstellung Absetzoption
Patientenperspektive
Ärztliche Perspektive
Gemeinsame Entscheidungsfindung
Weiter...
Absetzen ist eine Option!
Bei Ihnen haben sich über die Jahre viele Medikamente angesammelt. In so einer Situation sollte man nachschauen, was noch nötig ist und unnötige Medikamente absetzen.
Communicational hints in dependency of part and focus of the conversation
- Presenting options
- Eliciting patient's perspective
- Presenting professional perspective
- Shared decision

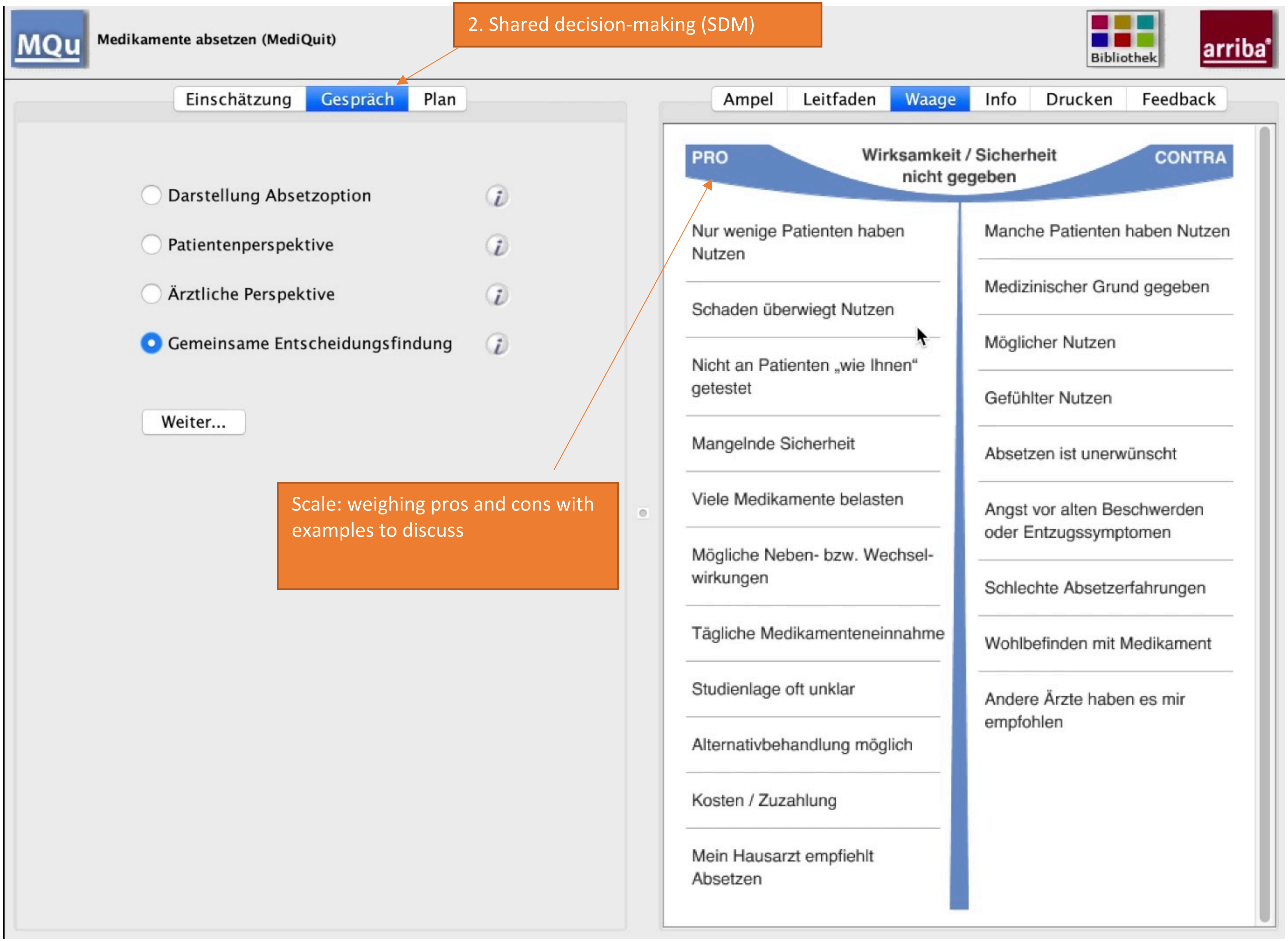

MQu
Medikamente absetzen (MediQuit)
2. Shared decision-making (SDM)
Bibliothek
arriba
Einschätzung
Gespräch
Plan
Darstellung Absetzoption
Patientenperspektive
Ärztliche Perspektive
Gemeinsame Entscheidungsfindung
Weiter...
Scale: weighing pros and cons with examples to discuss
Ampel
Leitfaden
Waage
Info
Drucken
Feedback
PRO
Wirksamkeit / Sicherheit nicht gegeben
CONTRA
Nur wenige Patienten haben Nutzen
Schaden überwiegt Nutzen
Nicht an Patienten „wie Ihnen“ getestet
Mangelnde Sicherheit
Viele Medikamente belasten
Mögliche Neben- bzw. Wechsel-wirkungen
Tägliche Medikamenteneinnahme
Studienlage oft unklar
Alternativbehandlung möglich
Kosten / Zuzahlung
Mein Hausarzt empfiehlt Absetzen
Manche Patienten haben Nutzen
Medizinischer Grund gegeben
Möglicher Nutzen
Gefühlter Nutzen
Absetzen ist unerwünscht
Angst vor alten Beschwerden oder Entzugssymptomen
Schlechte Absetzerfahrungen
Wohlbefinden mit Medikament
Andere Ärzte haben es mir empfohlen

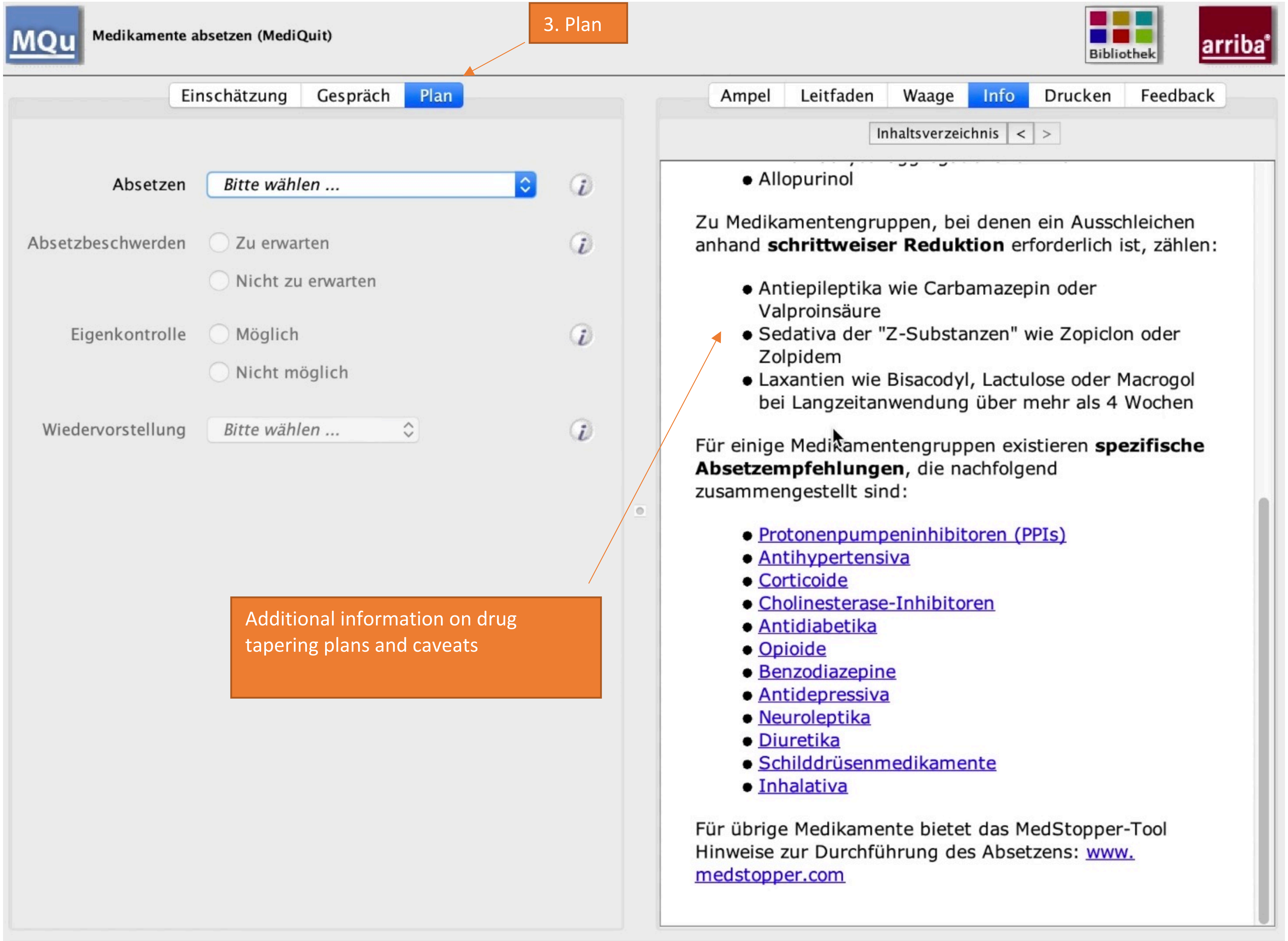

3. Plan
MQu
Medikamente absetzen (MediQuit)
Bibliothek
arriba
Einschätzung
Gespräch
Plan
Absetzen
Bitte wählen ...
Absetzbeschwerden
Zu erwarten
Nicht zu erwarten
Eigenkontrolle
Möglich
Nicht möglich
Wiedervorstellung
Bitte wählen ...
Additional information on drug tapering plans and caveats
Ampel
Leitfaden
Waage
Info
Drucken
Feedback
Inhaltsverzeichnis
Allopurinol
Zu Medikamentengruppen, bei denen ein Ausschleichen anhand schrittweiser Reduktion erforderlich ist, zählen:
Antiepileptika wie Carbamazepin oder Valproinsäure
Sedativa der "Z-Substanzen" wie Zopiclon oder Zolpidem
Laxantien wie Bisacodyl, Lactulose oder Macrogol bei Langzeitanwendung über mehr als 4 Wochen
Für einige Medikamentengruppen existieren spezifische Absetzempfehlungen, die nachfolgend zusammengestellt sind:
Protonenpumpeninhibitoren (PPIs)
Antihypertensiva
Corticoide
Cholinesterase-Inhibitoren
Antidiabetika
Opioide
Benzodiazepine
Antidepressiva
Neuroleptika
Diuretika
Schilddrüsenmedikamente
Inhalativa
Für übrige Medikamente bietet das MedStopper-Tool Hinweise zur Durchführung des Absetzens: www.medstopper.com

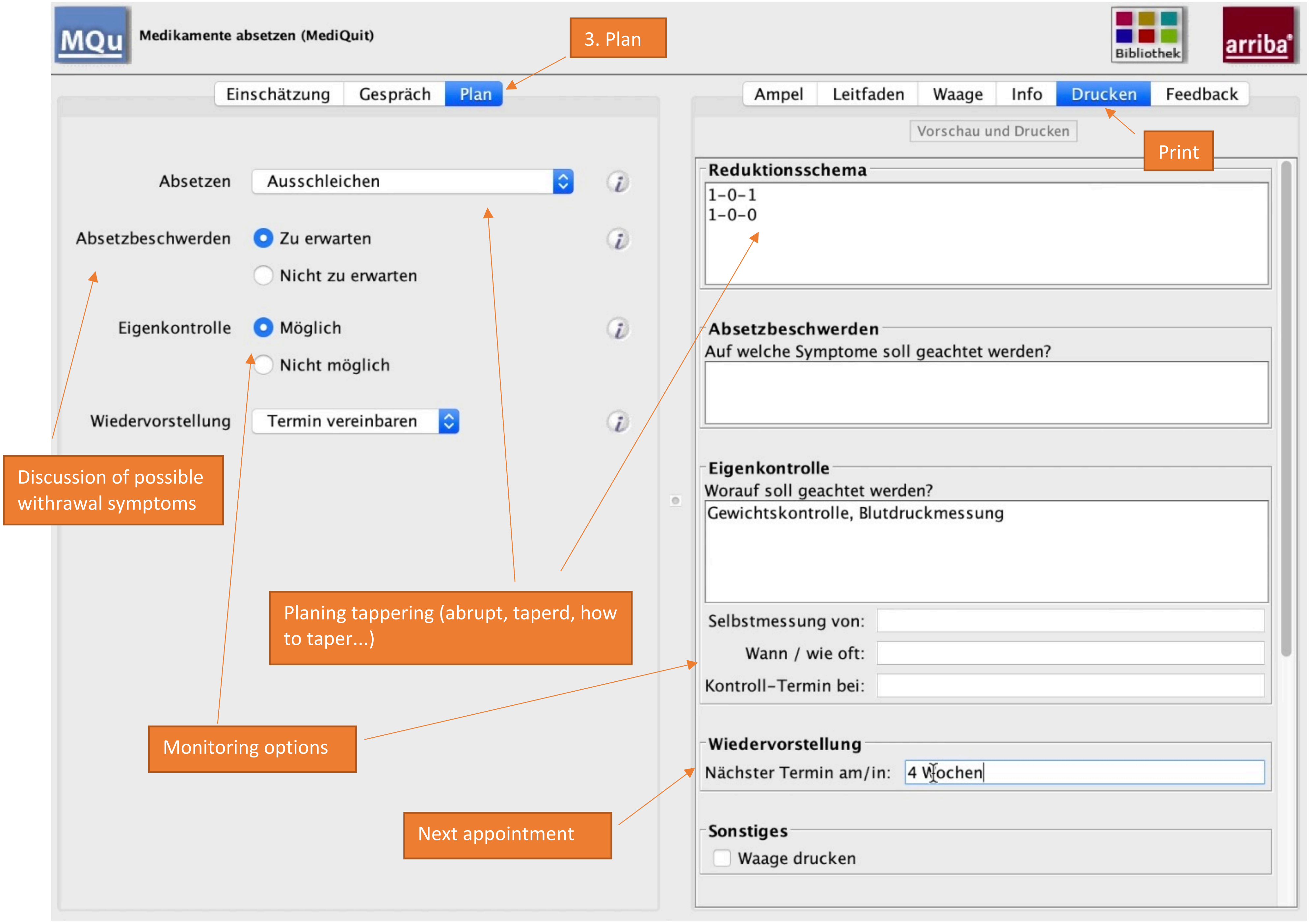
MQu
Medikamente absetzen (MediQuit)
3. Plan
Bibliothek
arriba
Einschätzung
Gespräch
Plan
Absetzen
Ausschleichen
Absetzbeschwerden
Zu erwarten
Nicht zu erwarten
Eigenkontrolle
Möglich
Nicht möglich
Wiedervorstellung
Termin vereinbaren
Discussion of possible withrawal symptoms
Planing tappering (abrupt, taperd, how to taper...)
Monitoring options
Next appointment
Ampel
Leitfaden
Waage
Info
Drucken
Feedback
Vorschau und Drucken
Print
Reduktionsschema
1-0-1
1-0-0
Absetzbeschwerden
Auf welche Symptome soll geachtet werden?
Eigenkontrolle
Worauf soll geachtet werden?
Gewichtskontrolle, Blutdruckmessung
Selbstmessung von:
Wann / wie oft:
Kontroll-Termin bei:
Wiedervorstellung
Nächster Termin am/in:
4 Wochen
Sonstiges
Waage drucken
